# Non-ST-elevation acute coronary syndromes with previous coronary artery bypass grafting: a meta-analysis of invasive vs. conservative management

**DOI:** 10.1093/eurheartj/ehae245

**Published:** 2024-05-28

**Authors:** Matthew Kelham, Rohan Vyas, Rohini Ramaseshan, Krishnaraj Rathod, Robbert J de Winter, Ruben W de Winter, Bjorn Bendz, Holger Thiele, Geir Hirlekar, Nuccia Morici, Aung Myat, Lampros K Michalis, Juan Sanchis, Vijay Kunadian, Colin Berry, Anthony Mathur, Daniel A Jones

**Affiliations:** Centre for Cardiovascular Medicine and Devices, Faculty of Medicine & Dentistry, Queen Mary University of London, London, UK; Barts Interventional Group, Barts Heart Centre, Barts Health NHS Trust, West Smithfield, London, UK; Barts Interventional Group, Barts Heart Centre, Barts Health NHS Trust, West Smithfield, London, UK; Centre for Cardiovascular Medicine and Devices, Faculty of Medicine & Dentistry, Queen Mary University of London, London, UK; Barts Interventional Group, Barts Heart Centre, Barts Health NHS Trust, West Smithfield, London, UK; Centre for Cardiovascular Medicine and Devices, Faculty of Medicine & Dentistry, Queen Mary University of London, London, UK; Barts Interventional Group, Barts Heart Centre, Barts Health NHS Trust, West Smithfield, London, UK; Department of Cardiology Heart Center, Amsterdam UMC, Universiteit van Amsterdam, Amsterdam, The Netherlands; Department of Cardiology, Amsterdam UMC, Vrije Universiteit Amsterdam, Amsterdam, The Netherlands; Department of Cardiology, Oslo University Hospital and Institute of Clinical Medicine, University of Oslo, Oslo, Norway; Heart Center Leipzig at University of Leipzig and Leipzig Heart Science, Leipzig, Germany; Department of Cardiology, Sahlgrenska University Hospital, Gothenburg, Sweden; IRCCS S. Maria Nascente—Fondazione Don Carlo Gnocchi ONLUS, Milan, Italy; Medical Director (Cardiology), Medpace UK, London, UK; 2nd Department of Cardiology, Faculty of Medicine, School of Health Sciences, University of Ioannina and University Hospital of Ioannina, University Campus, Ioannina 45110, Greece; Cardiology Department, University Clinic Hospital of València, INCLIVA University of València, CIBER CV, València, Spain; Cardiothoracic Centre, Freeman Hospital, Newcastle upon Tyne Hospitals NHS Foundation Trust and Translational and Clinical Research Institute, Faculty of Medical Sciences, Newcastle University, Newcastle upon Tyne, UK; British Heart Foundation Cardiovascular Research Centre, University of Glasgow, Glasgow, UK; Centre for Cardiovascular Medicine and Devices, Faculty of Medicine & Dentistry, Queen Mary University of London, London, UK; Barts Interventional Group, Barts Heart Centre, Barts Health NHS Trust, West Smithfield, London, UK; NIHR Barts Biomedical Research Centre, Queen Mary University of London, Charterhouse Square, London, UK; Centre for Cardiovascular Medicine and Devices, Faculty of Medicine & Dentistry, Queen Mary University of London, London, UK; Barts Interventional Group, Barts Heart Centre, Barts Health NHS Trust, West Smithfield, London, UK; NIHR Barts Biomedical Research Centre, Queen Mary University of London, Charterhouse Square, London, UK

**Keywords:** Ischaemic heart disease, Coronary artery bypass grafting, Non-ST-elevation acute coronary syndrome, Invasive coronary angiography, Conservative

## Abstract

**Background and Aims:**

A routine invasive strategy is recommended in the management of higher risk patients with non-ST-elevation acute coronary syndromes (NSTE-ACSs). However, patients with previous coronary artery bypass graft (CABG) surgery were excluded from key trials that informed these guidelines. Thus, the benefit of a routine invasive strategy is less certain in this specific subgroup.

**Methods:**

A systematic review and meta-analysis of randomized controlled trials (RCTs) was conducted. A comprehensive search was performed of PubMed, EMBASE, Cochrane, and ClinicalTrials.gov. Eligible studies were RCTs of routine invasive vs. a conservative or selective invasive strategy in patients presenting with NSTE-ACS that included patients with previous CABG. Summary data were collected from the authors of each trial if not previously published. Outcomes assessed were all-cause mortality, cardiac mortality, myocardial infarction, and cardiac-related hospitalization. Using a random-effects model, risk ratios (RRs) with 95% confidence intervals (CIs) were calculated.

**Results:**

Summary data were obtained from 11 RCTs, including previously unpublished subgroup outcomes of nine trials, comprising 897 patients with previous CABG (477 routine invasive, 420 conservative/selective invasive) followed up for a weighted mean of 2.0 (range 0.5–10) years. A routine invasive strategy did not reduce all-cause mortality (RR 1.12, 95% CI 0.97–1.29), cardiac mortality (RR 1.05, 95% CI 0.70–1.58), myocardial infarction (RR 0.90, 95% CI 0.65–1.23), or cardiac-related hospitalization (RR 1.05, 95% CI 0.78–1.40).

**Conclusions:**

This is the first meta-analysis assessing the effect of a routine invasive strategy in patients with prior CABG who present with NSTE-ACS. The results confirm the under-representation of this patient group in RCTs of invasive management in NSTE-ACS and suggest that there is no benefit to a routine invasive strategy compared to a conservative approach with regard to major adverse cardiac events. These findings should be validated in an adequately powered RCT.

## Structured Graphical Abstract

**Structured Graphical Abstract:**
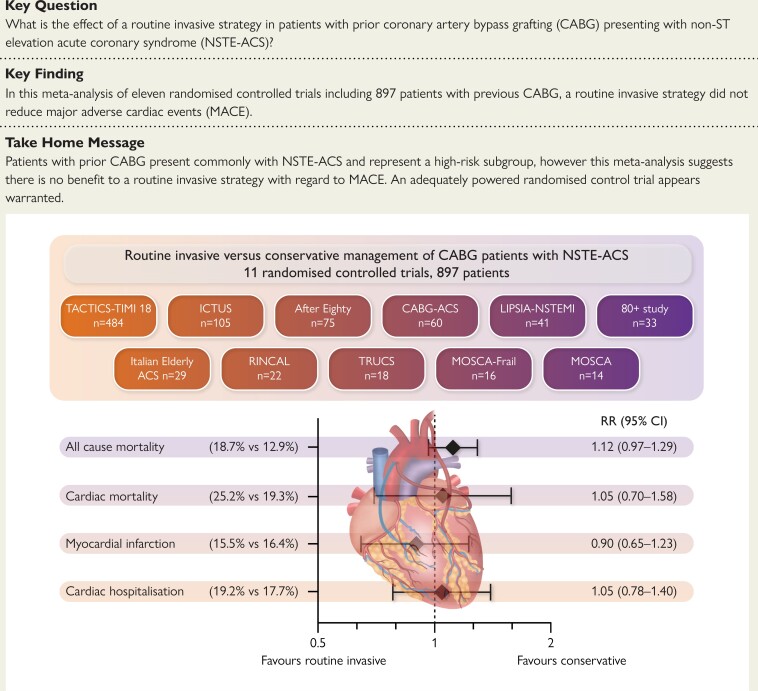
Summary of trials included and effect on major adverse cardiac events of a routine invasive vs. conservative approach to non-ST-elevation acute coronary syndrome (NSTE-ACS) in patients with prior coronary artery bypass grafting (CABG). CI, confidence interval; RR, risk ratio.

**See the editorial comment for this article ‘Non-ST-elevation acute coronary syndromes with previous coronary artery bypass grafting: is a routine invasive strategy needed?’, by F.J. Beerkens and D.L. Bhatt, https://doi.org/10.1093/eurheartj/ehae287.**

## Introduction

A routine invasive strategy is recommended in the management of higher risk patients with non-ST-elevation acute coronary syndrome (NSTE-ACS).^1^ This is based on the results of multiple randomized controlled trials (RCTs) that have compared a routine invasive with a conservative or selective invasive strategy, including over 11 000 patients, pooled in several meta-analyses.^2–6^ Patients with previous coronary artery bypass graft (CABG) surgery represent ∼10% of patients presenting with NSTE-ACS and represent a high-risk subgroup as they are older, with more comorbidities, and increased mortality compared to those without prior CABG.^7^ Of note, patients with previous CABG were excluded from some of the pivotal trials that informed NSTE-ACS guidelines (TIMI IIIB, FRISC II, RITA 3), thus the benefit of a routine invasive strategy is less certain in this group.^8–10^

Observational data consistently report that patients with prior CABG who present with ACS are less likely to undergo angiography or subsequent percutaneous coronary intervention (PCI).^11–13^ This likely reflects the higher rate of comorbidity, but also the risk of coronary angiography is greater in patients with previous CABG due to increased number of vessels to engage, variable location of bypass graft ostia, and often incomplete information available regarding the number and type of grafts placed.^14^ In addition, decisions regarding target vessel revascularization are more complex in CABG patients due to advanced atherosclerotic disease in native vessels and increased risk of distal embolization with vein graft PCI.^15^

The effect of a routine invasive approach in other high-risk subgroups of NSTE-ACS patients, such as older adults or those with chronic kidney disease, has been assessed in meta-analyses highlighting that the benefit of a routine invasive approach for NSTE-ACS may not apply in these groups and require dedicated RCTs.^16,17^ To our knowledge, the benefit of a routine invasive strategy in NSTE-ACS in patients with prior CABG has not been assessed. We therefore performed a systematic review and meta-analysis of RCTs to determine whether a routine invasive approach was superior to a selective invasive or conservative strategy in patients with prior CABG presenting with NSTE-ACS.

## Methods

### Search strategy and selection criteria

The systematic review was prospectively registered on the Prospero registry (CRD42022332048) and reported in accordance with the Preferred Reporting Items for Systematic Reviews and Meta-analyses guidelines.^18^ The Medline and EMBASE (Excerpta Medica database) databases were searched via National Institute for Health and Care Excellence’s Healthcare Database Advanced Search tool using a broad/sensitive approach, an identical strategy was used to search the Cochrane Library. Free-text searches for ‘acute coronary syndrome’ or ‘non ST elevation myocardial infarction’ or ‘unstable angina’ and ‘treat’ or ‘invasive’ or ‘conservative’ (plus synonyms) within the titles and abstracts of all records were combined with the appropriate Medical Subject Headings or EMBASE subject headings terms. Keywords using Medical Subject Headings/EMBASE subject headings where available included ‘Acute Coronary Syndrome’, ‘Non-ST Elevated Myocardial Infarction’, and ‘Angina, Unstable’. Results were limited to RCTs published from 1992 to May 2022 in English language. A search of all articles within ClinicalTrials.gov was conducted and in addition, a search was made of the PROSPERO registry for related meta-analysis. Reference lists of eligible articles were reviewed for further potential citations. Results were de-duplicated in EndNote (Thompson Reuters) reference management software and manually screened. The search strategy is detailed in the supplementary data.

Studies were deemed eligible if they met the following criteria: (i) RCTs assessing a routine invasive vs. conservative/selective invasive approach in NSTE-ACS; (ii) included patients with previous CABG; and (iii) patients with CABG were randomized to both arms. We excluded trials that included patients with ST-elevation myocardial infarction or excluded patients with prior CABG. The outcomes of interest were (i) all-cause mortality; (ii) cardiac mortality; (iii) myocardial infarction (MI); and (iv) cardiac-related hospitalization. Myocardial infarction and cardiac hospitalization events used the definition per respective RCT. Events at longest available follow-up were abstracted. As a *post hoc* analysis outcomes were assessed for non-CABG patients from the included trials.

Two investigators (M.K. and R.V.) independently assessed the eligibility of the studies for inclusion, any discrepancies were resolved by consensus after discussion with the senior investigator (D.A.J.). From the included studies, two reviewers (M.K. and D.A.J.) extracted demographic data and clinical outcomes using pre-specified data extraction forms. In cases where trials included patients with previous CABG but subgroup analysis was not published (either in the original publication or subsequent subgroup analysis publication), the trial corresponding author was contacted to request summary data for CABG patients (M.K. and D.A.J.). For each trial, risk of bias was independently assessed by two investigators (K.R. and R.R.) using the revised Cochrane RoB2 tool.^19^

### Data synthesis

Baseline categorical data from the included trials are summarized using weighted means and percentages. Trial-level data were analysed according to the intention-to-treat principle. We calculated pooled risk ratios (RRs) with 95% confidence intervals (CIs) for the outcomes of interest using the DerSimonian and Laird random-effects model with heterogeneity estimated from the Mantel–Haenszel method. Visual assessment of the forest plot and the *I*^2^ statistic were used to assess heterogeneity. Fixed-effects models (Mantel–Haenszel method) were performed as sensitivity analyses in the absence of high heterogeneity. In addition, further sensitivity analyses were conducted: excluding trials that only recruited older patients and conversely only including trials of older patients; of trials published prior to and post-2012. Publication bias was assessed with visual inspection of funnel plots and Egger’s test. For summary estimates, a *P* ≤ .05 (two-tailed) was considered significant. Analysis was conducted in Review Manager 5.4.

## Results

### Study selection and patient population

**Figure 1:**
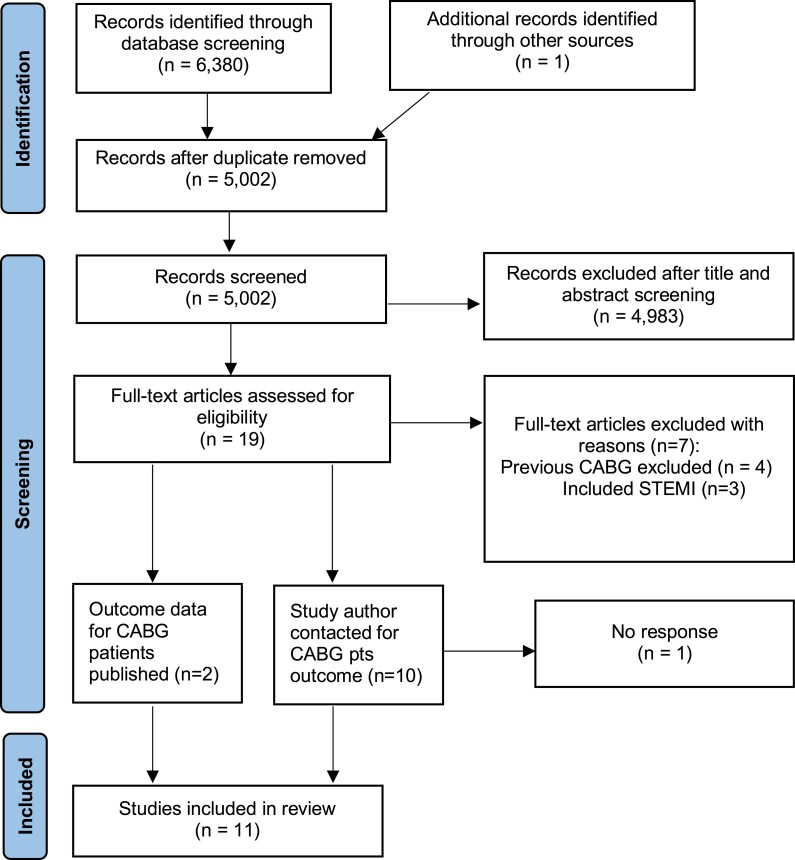
Study selection

A total of 19 trials assessing the effect of a routine invasive approach in NSTE-ACS were identified, with the full PRISMA flow diagram shown in *Figure 1*. Four trials (including 5871 patients) excluded patients with prior CABG,^8–10,20^ whilst three (including 1209 patients) included patients with ST-segment elevation^21–23^ leaving 12 eligible studies. Of these 12 trials (*n* = 5894 patients), 905 patients (15.4%) had a history of previous CABG. Data were obtained from 11 trials involving a total of 897 patients with NSTE-ACS randomly allocated to a routine invasive strategy (*n* = 477) or conservative/selective invasive (*n* = 420) strategy.^24–34^ One trial (CABG-ACS) had involved only patients with previous CABG, whilst TACTICS-TIMI 18 had published subgroup analysis of CABG patients.^28,35^ The remaining individual trial data for patients with prior CABG were obtained by contacting the trial corresponding author. The characteristics of the included trials and their overall results are presented in *Table 1*. In LIPSIA-NSTEMI, patients were randomized 1:1:1 between immediate invasive, early invasive, or selective invasive strategies; for the purpose of this meta-analysis, the immediate and early invasive group outcomes were pooled and compared to selective invasive. The baseline demographics and invasive management strategies for CABG patients from the trials are presented in *Table 2.* Six trials included elderly patients only with a mean age across the trials for CABG patients of 69.3 years. Inpatient angiography was performed in 97.4% of patients managed with a routine invasive strategy compared to 41.3% of patients allocated to a conservative/selective invasive strategy with percutaneous coronary intervention in 45.4% and 19.3%, respectively. The indications for angiography in the conservative/selective invasive group are highlighted in *Table 1*. CABG patient outcomes were available from a weighted mean of 2.0 (range 0.5–10) years follow-up. All trials were assessed as low risk of bias in all domains for the all-cause mortality outcome, with some concerns for the potentially subjective endpoints in MOSCA as these were not assessed by a blinded endpoint committee. The risk of bias assessments are reported in Supplementary data online, *Tables S1* and *S2*.

**Table 1:** Characteristics of included studies

Study	Total patients	Patients with prior CABG	Study population	Treatment arms	Primary endpoint (and key secondary)	Trial findings
Cannon 2001-(TACTICS-TIMI 18)	2220	484	Over 18 years, unstable angina and NSTEMI patients with ECG changes, elevated cardiac markers, or a history of coronary artery disease International Multicentre (180 sites), recruiting 1997–99	1:1 randomization Invasive: angiography between 4 and 48 h ± revascularization when appropriate Conservative: angiography only if objective evidence of recurrent ischaemia or an abnormal stress test	Combined incidence of death, non-fatal MI, and rehospitalization for ACS at 6 months	Early invasive strategy significantly reduced the incidence of major cardiac events (OR 0.78, 95% CI 0.62–0.97, *P* = .025) Kugelmass 2006: (substudy of CABG patients in TACTICS-TIMI 18) Early invasive strategy reduces risk of MI (OR 0.44, 95% CI 0.21–0.93, *P* = .032)
De Belder 2021 (RINCAL)	251	22	Over 80 years, admitted with chest pain, ischaemic ECG changes, and elevated troponin (NSTEMI) 12 UK intervention centres, 2014–18	1:1 randomization Intervention guided: angiography ± PCI or CABG OMT alone: angiography if ongoing chest pain with or without dynamic ECG changes and/or troponin rise	Composite of all-cause mortality and non-fatal myocardial infarction at 1 year	No difference in the primary endpoint (18.5% vs. 22.2%, *P* = .39)
De Winter 2005 (ICTUS)	1200	105	18–80 years, ACS without ST-elevation with chest pain, elevated troponin, and either ECG changes of ischaemia or documented history of coronary disease 42 Dutch Hospitals (12 high volume centres), 2001–03	1:1 randomization Early invasive: angiography within 24–48 h and PCI or CABG when appropriate Selective invasive: angiography only if refractory angina despite OMT or documented ischaemia	Composite of death, recurrent MI, of rehospitalization for angina	No difference in primary endpoint (22.7% vs. 21.2%, RR 1.07, 95% CI 0.87–1.33, *P* = .33)
Hirlekar 2020 (80+ study)	186	33	Over 80 years with NSTE-ACS 3 Swedish Hospitals 2009–17	1:1 randomization Invasive: angiography ± PCI or CABG Conservative: angiography only if refractory chest pain, haemodynamic instability, heart failure, or life-threatening cardiac arrhythmia	Combined endpoint of major adverse cardiac and cerebrovascular events (MACCE)	No significant difference in primary endpoint (33.3% vs. 36.6%, HR 0.9, 95% CI 0.55–1.46, *P* = .66)
Lee 2019 (CABG-ACS)	60	60	Over 18 years. Unstable angina or NSTEMI; stabilized symptoms without recurrent chest pain; prior CABG 4 UK hospitals 2012–13	1:1 randomization Invasive: angiography within 72 h Medical: angiography if recurrent/refractory angina with ECG changes, new ST-elevation, heart failure secondary to myocardial ischaemia	Adherence with randomized strategy at 30 days Secondary outcome: MACE (all-cause mortality, rehospitalization for refractory ischaemia/angina, MI, or hospitalization for heart failure)	One crossover of medical group to invasive management No difference in composite MACE (42% vs. 45%, HR 0.85, 95% CI 0.39–1.83)
Michalis 2000 (TRUCS)	148	18	Adult patients with refractory unstable angina without MI or death within 48 h of admission 2 Greek District Hospitals without on-site cardiac surgery 1997–98	1:1 randomization Invasive: angiography on day of diagnosis of refractory angina Conservative: angiography if refractory ischaemia for 5 days	In-hospital stabilization (not having MI or death during admission), new non-fatal MI and death, duration of hospitalization	In-hospital: non-fatal MI 2.6% vs. 4.2%, *P* = ns Death 1.3% vs. 8.3%, *P* = .046 12 months: non-fatal MI 3.9% vs. 4.2%, *P* = ns Death 3.9% vs. 12.5%, *P* = .053
Sanchis 2016 (MOSCA)	106	14	Over 70 years, NSTEMI and with comorbidities (at least two of peripheral artery disease, cerebral vascular disease, dementia, chronic pulmonary disease, chronic renal failure, or anaemia) 6 Spanish Hospitals 2012–14	1:1 randomization Invasive: angiography within 72 h Conservative: angiography if recurrent ischaemia or heart failure, or in case of positive pre-discharge non-invasive stress test	Composite of all-cause mortality, re-infarction, and readmission for cardiac cause	No difference in primary endpoint (IRR 0.946, 95% CI 0.466–1.918, *P* = .877) at long-term
Sanchis 2023 (MOSCA-FRAIL)	167	16	Over 70 years with Clinical Frailty Score ≥ 4 and NSTEMI 13 Spanish Hospitals 2017–21	1:1 Randomization Routine invasive: angiography within 72 h Conservative: angiography in case of recurrent ischaemia	Number of days alive and out of hospital from discharge to 1 year (DAOH) Co-primary: composite of cardiac death, re-infarction, or post-discharge revascularization	Non-significant increase in DAOH in conservative managed group (284 vs. 312 days, *P* = .12) No difference in co-primary endpoint of ischaemic cardiac events (HR 0.92, 95% CI 0.54–1.57, *P* = .78)
Savonitto 2012 (Italian Elderly ACS)	313	29	Over 75 years with NSTE-ACS within 48 h of symptoms 21 Italian Hospitals 2008–10	1:1 randomization Early angiography: angiography within 72 h Initially conservative: angiography in case of refractory ischaemia, myocardial (re)infarction, heart failure of ischaemic origin, or malignant ventricular arrhythmias	Composite of death, myocardial infarction, disabling stroke, and repeat hospital stay for cardiovascular causes or severe	No difference in primary outcome (27.9% vs. 34.6%, HR 0.8, 95% CI 0.53–1.19, *P* = .26)
Tegn 2016 (After Eighty Study)	457	75	Over 80 years with NSTEMI or unstable angina 16 Norwegian Hospitals 2010–14	1:1 Randomization Invasive: coronary angiography day after randomization Conservative: considered for angiography if had re-infarction, refractory angina, malignant ventricular arrhythmias, or increasing symptoms of heart failure	Composite of myocardial infarction, need for urgent revascularization, stroke, and death	Reduction of composite events in routine invasive group at 1.5 years (40.6% vs. 61.4%, HR 0.53, 95% CI 0.41–0.69, *P* = .0001)
Thiele 2012 (LIPSIA-NSTEMI)	602	41	18–90 years with NSTEMI with ischaemic symptoms < 24 h before randomization and elevated troponin Six German hospitals with 24 h PCI facilities 2006–09	1:1:1 randomization Immediate invasive: angiography within 2 h of randomization Early invasive: angiography on next working day Selective invasive: underwent angiography if refractory angina, new ST-elevation, T wave inversion > 3 mm, dynamic ST depression, development of rhythmic instability or refractory heart failure, reduced LVEF < 45%, clinically significant ischaemia on a pre-discharge exercise test	Primary outcome: peak CK-MB during index hospitalization Secondary endpoint: composite of (i) death and non-fatal MI; (ii) death, non-fatal MI, and refractory ischaemia; (iii) death, non-fatal MI, refractory ischaemia, and rehospitalization for unstable angina within 6 months	No significant difference in peak CK-MB. No difference in key secondary outcomes: death and infarction (21.0% vs. 16.0% vs. 14.5%, *P* = .17)

ACS, acute coronary syndrome; CABG, coronary artery bypass grafting; CK-MB, creatine kinase-myocardial band; MI, myocardial infarction; OMT, optimal medical therapy; PCI, percutaneous coronary intervention.

**Table 2:** Baseline demographics and invasive management strategies of coronary artery bypass grafting patients in included studies

Study	Mean age, years	Male	Diabetes	Troponin positive	Angiography during index admission		PCI during index admission		CABG during index admission		Follow-up^a^
					Routine invasive	Conservative	Routine invasive	Conservative	Routine invasive	Conservative	
Cannon 2001-(TACTICS-TIMI 18)^b^	64.2	74.0%	33.1%	40.3%	97.4%	50.7%	37.4%	22.2%	8.9%	6.3%	6 months
De Belder 2021 (RINCAL)	84.1	68.2%	13.6%	100.0%	83.3%	20.0%	41.7%	10.0%	0.0%	0.0%	1 year
De Winter 2005 (ICTUS)	66.4	78.1%	27.6%	100.0%	98.4%	53.5%	59.7%	27.9%	8.1%	0.0%	5 years
Hirlekar 2020 (80+ study)	84.4	66.7%	30.3%	97.0%	100%	0.0%	57.9%	0.0%	0.0%	0.0%	1 year
Lee 2019 (CABG-ACS)	70.9	71.7%	35.0%	68.3%	100%	0.0%	32.3%	0.0%	0.0%	0.0%	2 years
Michalis 2000 (TRUCS)^b,c^	62.5	73.0%	28.4%	0.0%	100%	52.8%	52.6%	31.9%	25.0%	5.6%	1 year
Sanchis 2016 (MOSCA)	80.3	92.9%	50.0%	100.0%	100.0%	25.0%	60.0%	0.0%	0.0%	0.0%	2.5 years
Sanchis 2023 (MOSCA-FRAIL)	84.7	62.5%	56.3%	100.0%	100.0%	27.3%	20.0%	27.3%	0.0%	0.0%	1 year
Savonitto 2012 (Italian Elderly ACS)	79.4	72.4%	44.8%	58.6%	88.2%	33.3%	52.9%	16.7%	0.0%	0.0%	1 year
Tegn 2016 (After Eighty Study)	84.5	72.0%	28.0%	92.0%	97.7%	0.0%	53.5%	0.0%	14.0%	0.0%	10 years
Thiele 2012 (LIPSIA-NSTEMI)	73.2	80.5%	46.3%	100.0%	100.0%	93.8%	76.0%	50.0%	4.0%	0.0%	6 months
Weighted Mean	69.3	74.1%	33.1%	61.5%	97.4%	41.3%	45.4%	19.3%	7.2%	3.7%	2.0 years

^a^Refers to the time period for which outcomes data were available/supplied for CABG patients.

^b^Angiography percentage from overall trial cohort.

^c^Demographics of overall trial cohort.

### Mortality

**Figure 2:**
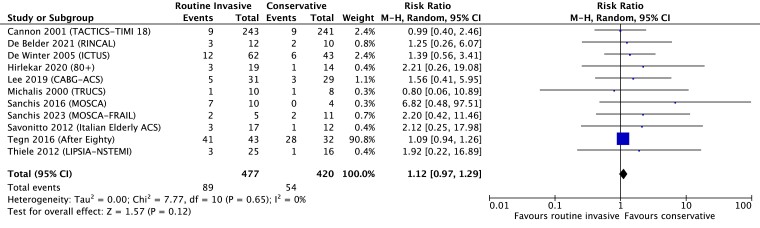
Effect on mortality of routine invasive vs. conservative strategy in CABG patients presenting with NSTE-ACS, random-effects model

All trials reported all-cause mortality as an endpoint. The total mortality follow-up varied from 6 months to 10 years. Heterogeneity was assessed as low (*I*^2^ = 0%). Overall, 89 of 477 patients (18.7%) randomized to a routine invasive strategy died during follow-up vs. 54 of 420 patients (12.9%) randomized to a conservative strategy. A routine invasive strategy did not reduce all-cause mortality compared with a conservative strategy: RR 1.12 (95% CI 0.97–1.29), *P* = .12 (random-effects model, *Figure 2*), although a fixed-effect model suggested a reduction in mortality with a conservative approach: RR 1.28 (95% CI 1.01–1.63), *P* = .04 (see Supplementary data online, *Figure S1*). Sensitivity analysis excluding older patient-only trials [RR 1.25 (0.72–2.15), *P* = .42] or trials only including older patients [RR 1.57 (0.76–3.25), *P* = .23] produced similar results (see Supplementary data online, *Table S3*), as did sensitivity analysis of trials published pre- [RR 1.24 (0.7–2.21), *P* = .45] and post-2012 [RR 1.47 (0.78–2.76), *P* = .24] (see Supplementary data online, *Table S4*).

### Cardiac mortality

Seven trials, involving 257 patients, contributed to the analysis of cardiac mortality (definitions Supplementary data online, *Table S5*). Heterogeneity was assessed as low (*I*^2^ = 0%). Overall 36 of 143 patients (25.2%) managed with a routine invasive strategy vs. 22 of 114 (19.3%) managed with a conservative strategy were adjudged to have died of a cardiac condition. The risk of cardiac mortality did not differ significantly between strategies: RR 1.05 (95% CI 0.70–1.58), *P* = .81 (random-effects model, Supplementary data online, *Figure S2*). A fixed-effects model yielded comparable results: RR 1.22 (0.80–1.86), *P* = .36 (see Supplementary data online, *Figure S3*). Sensitivity analysis of excluding older patient-only trials [RR 2.29 (0.39–13.34), *P* = .36] or trials only including elderly patients [RR 1.14 (0.74–1.75), *P* = .55] produced similar results (see Supplementary data online, *Table S3*) as did sensitivity analysis of trials published pre- [RR 1.68 (0.35–8.09), *P* = .52] and post-2012 [RR 1.02 (0.67–1.55), *P* = .94] (see Supplementary data online, *Table S4*).

### Myocardial infarction

**Figure 3:**
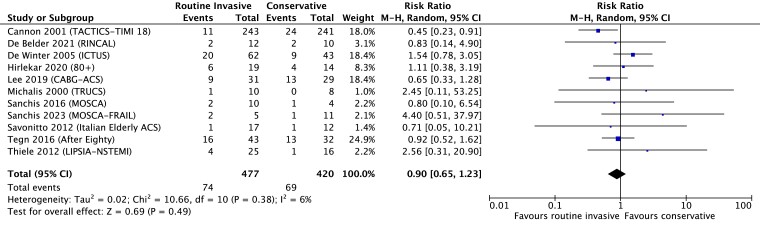
Effect on myocardial infarction of routine invasive vs. conservative strategy in CABG patients presenting with NSTE-ACS, random-effects model

All trials reported MI as an endpoint, with slight variation of the contemporary definition between studies (see Supplementary data online, *Table S6*).^36–38^ Overall, 74 of 477 patients (15.5%) managed with a routine invasive strategy vs. 69 of 420 (16.4%) managed with a conservative strategy experienced an MI. Heterogeneity was assessed as low (*I*^2^ = 6%). The risk of MI did not differ between groups: RR 0.90 (95% CI 0.65–1.23), *P* = .49 (random-effects model, *Figure 3*). A fixed-effects model yielded comparable results: RR 0.88 (0.66–1.17), *P* = .38 (see Supplementary data online, *Figure S4*). Sensitivity analysis excluding older patient-only trials [RR 0.88 (0.47–1.66), *P* = .70] or trials only including older patients [RR 1.00 (0.63–1.57), *P* = .99] produced similar results (see Supplementary data online, *Table S3*) as did sensitivity analysis of trials published pre- [RR 1.00 (0.45–2.25), *P* = .99] and post-2012 [RR 0.88 (0.60–1.28), *P* = .50] (see Supplementary data online, *Table S4*).

Death or MI was assessed as a combined endpoint and was present in 143 (30%) patients managed with a routine invasive strategy vs. 106 (25.2%) patients with a conservative strategy. Heterogeneity was low (*I*^2^ = 27%). The risk did not differ between groups with a random-effects [RR 1.12 (0.89–1.40), *P* = .35, Supplementary data online, *Figure S5*] or fixed-effects model [RR 1.08 (0.90–1.30), *P* = .39].

### Cardiac hospitalizations

Nine trials, comprising 717 patients, reported outcomes for cardiac hospitalizations. The definition of cardiac hospitalization varied between studies (see Supplementary data online, *Table S7*). Overall, 74 of 372 patients (19.2%) managed with a routine invasive strategy vs. 61 of 345 (17.7%) managed with a conservative strategy had a further reported cardiac hospitalization following the index event during trial follow-up. Heterogeneity was assessed as low (*I*^2^ = 0%). The risk of cardiac hospitalization did not differ between groups: RR 1.05 (95% CI 0.78–1.40), *P* = .77 (random-effects model, Supplementary data online, *Figure S6*) with comparable results using a fixed-effects model [RR 1.08 (0.80–1.45), *P* = .61] (see Supplementary data online, *Figure S7*). Sensitivity analysis excluding older patient-only trials [RR 0.98 (0.69–1.40), *P* = .91] or trials only including older patients [RR 1.45 (0.85–2.47), *P* = .17] produced similar results (see Supplementary data online, *Table S3*) as did sensitivity analysis of trials published pre- [RR 1.01 (0.70–1.44), *P* = .97] and post-2012 [RR 1.31 (0.78–2.19), *P* = .31] (see Supplementary data online, *Table S4*).

### *Post hoc* analysis

To address the treatment effect with presence or absence of prior CABG, pooled risk ratios were calculated for all-cause mortality and MI, as the outcomes consistently reported, for non-CABG patients in the studies included (*n* = 4810) (see Supplementary data online, *Table S8*). For both outcomes, the RRs are lower (i.e. favouring a routine invasive strategy) when CABG patients are excluded compared to the CABG patient outcomes. When the outcomes of trials that excluded patients with prior CABG (TIMI IIIB, FRISC II, RITA 3, VINO) are also included (*n* = 10 681), these reduce further.^8–10,20^

## Discussion

This is the first meta-analysis of randomized trials assessing the effect of a routine invasive strategy in patients with prior CABG who present with NSTE-ACS. This confirms the under-representation of patients with previous CABG in RCTs of invasive management in NSTE-ACS, comprising only 7.7% of patients due to exclusion from the largest trials. Combining the outcomes data from 11 RCTs including 897 patients, there was no benefit to a routine invasive strategy with regard to all-cause or cardiac mortality, MI, or cardiac hospitalization in patients with previous CABG. In fact, all endpoints aside from recurrent MI were more frequent in the routine invasive arm (*Structured Graphical Abstract*). This directly calls into question the current recommendations of a routine invasive strategy in this patient cohort.

The results of this meta-analysis of RCT data differs from the body of observational evidence that appears to be in support of a routine invasive strategy in patients with prior CABG. In a cohort of over 10 000 patients with a history of CABG younger than 80 years old admitted with NSTE-ACS to coronary care units in Sweden between 1995 and 2004, revascularization (PCI or CABG) within 14 days of admission was associated with a marked reduction in 1-year mortality (RR 0.67, 95% CI 0.56–0.81).^39^ In the US National Inpatient Sample database of all MI admissions 2004–15, which included almost 450 000 prior CABG patients, lower adjusted in hospital all-cause mortality [odds ratio (OR) 0.45, 95% CI 0.38–0.53] and MACE were observed (OR 0.64, 95% CI 0.57–0.72) in prior CABG patients who underwent PCI compared with those managed medically.^11^ Similar results were observed in the UK from the Myocardial Infarction National Audit Project database, including over 25 000 patients with prior CABG between 2010 and 2017, with lower adjusted risk of inpatient mortality (OR 0.67, 95% CI 0.46–0.98), although similar rates of re-infarction (OR 1.13, 95% CI 0.81–1.57), observed in patients who underwent PCI compared to those managed medically.^12^ Although conversely, a subgroup analysis of patients with prior CABG from the ACUITY trial (*n* = 2475) reported significantly higher 30-day and 1-year MACE rates in ACS patients who underwent PCI compared to medical management.^40^ An important caveat to this observational data is that it compares patients who underwent revascularization compared to medical management rather than those who underwent a routine invasive strategy compared to a conservative/selective invasive approach as in the RCTs. Multiple factors will influence the clinical decision for both angiography and then subsequent revascularization including frailty, comorbidity, and lesion complexity. Whilst observational studies will attempt to adjust for some of these factors, namely age and comorbidity, there are likely unmeasured confounders that will influence outcomes. Included in this meta-analysis is the only previously published randomized evidence of the effect of a routine invasive strategy, which came from the TACTICS-TIMI 18 and CABG-ACS trials. TACTICS-TIMI 18 is the largest relevant trial and reported a significant reduction in MI at 6 months in those randomized to an early invasive strategy, whereas the CABG-ACS pilot study reported similar 2-year MACE outcomes.^28,35^

The lack of observed benefit for a routine invasive strategy amongst CABG patients with NSTE-ACS in this meta-analysis is likely multifactorial. Firstly, current guidelines recognize that a routine invasive approach is not proved by the current evidence to reduce all-cause mortality in the overall population of NSTE-ACS patients, therefore it is logical that a benefit in mortality is not seen in a smaller subset of patients.^1^ Of interest, when a fixed-effect model is used, there appears to be reduced mortality with a conservative approach. However, whilst heterogeneity was assessed as low by the *I*^2^ statistic, the populations of each study varied in age, timeframe, and demographics and one would expect the true effect size may vary study to study, therefore a random-effects model is preferable. Secondly, the trials included recruited patients over a period of 24 years, with 607/897 (68%) of patients enrolled prior to 2004.^24,26,29^ in which time there have been multiple improvements in invasive management such as radial access and drug eluting stents, although medical secondary prevention has also improved with respect to modern dual-antiplatelet therapy and intensive lipid lowering. Finally, six trials recruited older patients only, which may have contributed to reduced benefit from an invasive strategy, although we performed sensitivity analysis of the endpoints only including non-older patients’ trials with similar results. Our *post hoc* analysis assessing the outcomes of non-CABG patients from the included trials, and including NSTE-ACS trials that excluded CABG patients, supports our overall findings that the benefits of a routine invasive strategy may not apply to CABG patients as they do to non-CABG patients.

The benefit seen in observational studies of PCI in CABG patients compared to medical management will be subject to unmeasured confounders. In addition, in a routine invasive strategy, patients are exposed to the potential harm of performing angiography without necessarily receiving the benefit of having PCI (either because not felt to be required or not technically feasible in the setting of advanced and often calcific atherosclerotic disease). The rates of PCI in the routine invasive group are lower in CABG patients compared to the non-CABG patients of the included trials (45% vs. 53%, *P* = .002). Invasive angiography in patients with prior CABG takes longer, with higher radiation and contrast exposure and is known to be higher risk, particularly with regard to neurological complications and risk of contrast induced nephropathy.^14,41,42^ When revascularization is felt to be required in patients post-CABG, redo CABG is associated with two-four-fold increased mortality compared to first-time CABG and PCI to vein grafts is associated with an increased risk of distal embolization and periprocedural MI.^15,43^ Therefore, PCI to native vessels is recommended but not always possible to be performed.^15^ Our results highlight that even when managed with a routine invasive strategy, only 45% of patients receive PCI compared to 19% in the selective invasive arm, with this relatively small difference possibly explaining the divergence from the outcomes of observational data that compare those who have received PCI with those who have not. One potential consideration is the use of computed tomography coronary angiography (CTCA), which is known to have excellent sensitivity and specificity to assess bypass grafts, as the first line test in patients with prior CABG presenting with NSTE-ACS.^44,45^ In that situation, if grafts are found to be patent, then the higher risks of invasive angiography can be avoided, or it can help target the use of PCI, an approach which has recently been demonstrated to reduce MACE in the BYPASS-CTCA trial.^46^

### Limitations

Our meta-analysis includes outcomes data from 99% (897/905) of patients with prior CABG in trials that assessed a routine invasive approach in NSTE-ACS, with data not obtained from only one study including eight CABG patients.^47^ Despite obtaining outcomes data for the vast majority of CABG patients in relevant trials, this still only constitutes 897 patients across 11 trials. Therefore, our findings may simply reflect RCT data of CABG NSTE-ACS patients lacking statistical power to detect differences in MACE, with sensitivity analysis especially underpowered. In particular, the number of patients included in the analysis of cardiac mortality and cardiac hospitalization (the definition of which varied from study to study) limits the conclusions that can be drawn from them. As randomization in the individual studies was not stratified by CABG status, there may be differences in baseline risk profile that could impact on outcomes. Despite similar protocol indications for invasive angiography in the conservative/selective invasive groups of each study, predominantly recurrent ischaemia, there were wide variations in the incidence of inpatient angiography in this arm across the studies. This will have impacted our results compared to assessing outcomes of purely invasive vs. medical management. In addition, the majority of patients were enrolled over 20 years ago, since which both invasive management and medical secondary prevention have been refined and therefore outcomes may differ in a contemporary trial. Changes in CABG technique in this time may also influence outcomes, for example increased use of bilateral internal mammary arteries would have improved patency and are easier to evaluate with invasive angiography than multiple vein grafts. As discussed above, the more recent trials (excluding CABG-ACS) compared outcomes in older patients only, whose findings may not reflect the entire post-CABG patient population. Assessment of publication bias with funnel plots and Egger’s test (see Supplementary data online, *Figures S8*–*S11*, Supplementary data online, *Table S9*) suggested that publication bias was possible for the MI outcome (*P* = .057), although of note the overall number of trials was small and only two trials previously published CABG patient outcomes, thus formal assessment of publication bias was less relevant. For this meta-analysis, we did not obtain individual patient data (IPD) in order to perform an IPD meta-analysis, which may have enhanced our findings as would allow per-protocol analysis and assessment of impact of risk factors such as age. Finally, we reported outcomes of total events at the longest available follow-up time point, whilst there may be clinically meaningful benefits in short- or long-term reduction of ischaemic events not demonstrated with our analysis but which would be available if longer term follow-up (with events at different time points) was available.

## Conclusions

Patients with prior CABG present commonly with NSTE-ACS and represent a high-risk subgroup. Despite prior observational study evidence suggesting that there is a benefit of early revascularization, and TACTICS-TIMI 18 reporting a reduction in MI with a routine invasive approach, this meta-analysis of randomized trials suggests that there is no benefit to a routine invasive strategy compared to a conservative approach in this group. An adequately powered RCT appears warranted to further explore this finding.

## Supplementary Material

ehae245_Supplementary_Data

## Declarations

### Data Availability

Previously unpublished data collected for the meta-analysis could be made available to others with the support of the specific trials author.

### Pre-registered Clinical Trial Number

Prospero registration: CRD42022332048.
