# Supplementary material for: Non-ST-elevation acute coronary syndromes with previous coronary artery bypass grafting: a meta-analysis of invasive vs. conservative management
Source: Eur Heart J. 2024 May 28;45(27):2380–91. doi: 10.1093/eurheartj/ehae245 (PMC11242441; doi:10.1093/eurheartj/ehae245)
Supplement: ehae245_Supplementary_Data [file ehae245_supplementary_data.docx]

**CABG-NSTEACS Meta-Analysis Supplement**

**Contents**

Table S1: Risk of Bias for All-Cause Mortality - Page 2

Table S2: Risk of Bias for Cardiac Mortality, Myocardial Infarction, and Cardiac Hospitalisation – Page 3

Figure S1: Forest plot of Mortality, Fixed effect model-Page 3

Table S3: Sensitivity Analysis of Trials by Elderly Patient Status-Page 4

Table S4: Sensitivity Analysis of Trials by Publication Date – Page 4

Table S5: Cardiac Death Definitions – Page 4

Figure S2: Forest plot of Cardiac Mortality, Random effects model- Page 4

Figure S3: Forest plot of Cardiac Mortality, Fixed effect model-Page 5

Table S6: Myocardial Infarction Definitions-Page 5

Figure S4: Forest plots of Myocardial Infarction, fixed effects model-Page 6

Figure S5: Forest plot of Mortality or MI, Random effect model-Page 6

Table S7: Cardiac Hospitalisations Definitions-Page 6

Figure S6: Forest plot of Cardiac Hospitalisation, random effects model-Page 7

Figure S7: Forest plot of Cardiac Hospitalisation, fixed effects model-Page 7

Table S8: Non-CABG patient’s outcomes-Page 7

Figure S8: Funnel Plot for Meta-Analysis of Mortality-Page 8

Figure S9: Funnel Plot for Meta-Analysis of Cardiac Mortality-Page 8

Figure S10: Funnel Plot for Meta-Analysis of MI-Page 8

Figure S11: Funnel Plot for Meta-Analysis of Cardiac Hospitalisation-Page 8

Table S9: Egger’s Test for the Explored Outcomes-Page 9

Search Strategy-Cochrane Library-Page 9

Search Strategy-Embase and Medline-Page 9

Table S1: Risk of Bias for All-Cause Mortality

| **Study ID** | **D1** | **D2** | **D3** | **D4** | **D5** | **Overall** |  |  |  |
| --- | --- | --- | --- | --- | --- | --- | --- | --- | --- |
| TACTICS-TIMI 18 |  |  |  |  |  |  |  |  | Low risk |
| RINCAL |  |  |  |  |  |  |  |  | Some concerns |
| ICTUS |  |  |  |  |  |  |  |  | High risk |
| 80+ |  |  |  |  |  |  |  |  |  |
| CABG-ACS |  |  |  |  |  |  |  | D1 | Randomisation process |
| TRUCS |  |  |  |  |  |  |  | D2 | Deviations from the intended interventions |
| MOSCA |  |  |  |  |  |  |  | D3 | Missing outcome data |
| MOSCA-Frail |  |  |  |  |  |  |  | D4 | Measurement of the outcome |
| Italian Elderly ACS |  |  |  |  |  |  |  | D5 | Selection of the reported result |
| After Eighty |  |  |  |  |  |  |  |  |  |
| LIPSIA-NSTEMI |  |  |  |  |  |  |  |  |  |

Table S2: Risk of Bias for Cardiac Mortality*, Myocardial Infarction, and Cardiac Hospitalisation**

*Exclude TACTICS-TIMI, ICTUS, 80+, TRUCS

**Exclude ICTUS, TRUCS, After Eighty

| **Study ID** | **D1** | **D2** | **D3** | **D4** | **D5** | **Overall** |  |  |  |
| --- | --- | --- | --- | --- | --- | --- | --- | --- | --- |
| TACTICS-TIMI 18 |  |  |  |  |  |  |  |  | Low risk |
| RINCAL |  |  |  |  |  |  |  |  | Some concerns |
| ICTUS |  |  |  |  |  |  |  |  | High risk |
| 80+ |  |  |  |  |  |  |  |  |  |
| CABG-ACS |  |  |  |  |  |  |  | D1 | Randomisation process |
| TRUCS |  |  |  |  |  |  |  | D2 | Deviations from the intended interventions |
| MOSCA |  |  |  |  |  |  |  | D3 | Missing outcome data |
| MOSCA-Frail |  |  |  |  |  |  |  | D4 | Measurement of the outcome |
| Italian Elderly ACS |  |  |  |  |  |  |  | D5 | Selection of the reported result |
| After Eighty |  |  |  |  |  |  |  |  |  |
| LIPSIA-NSTEMI |  |  |  |  |  |  |  |  |  |


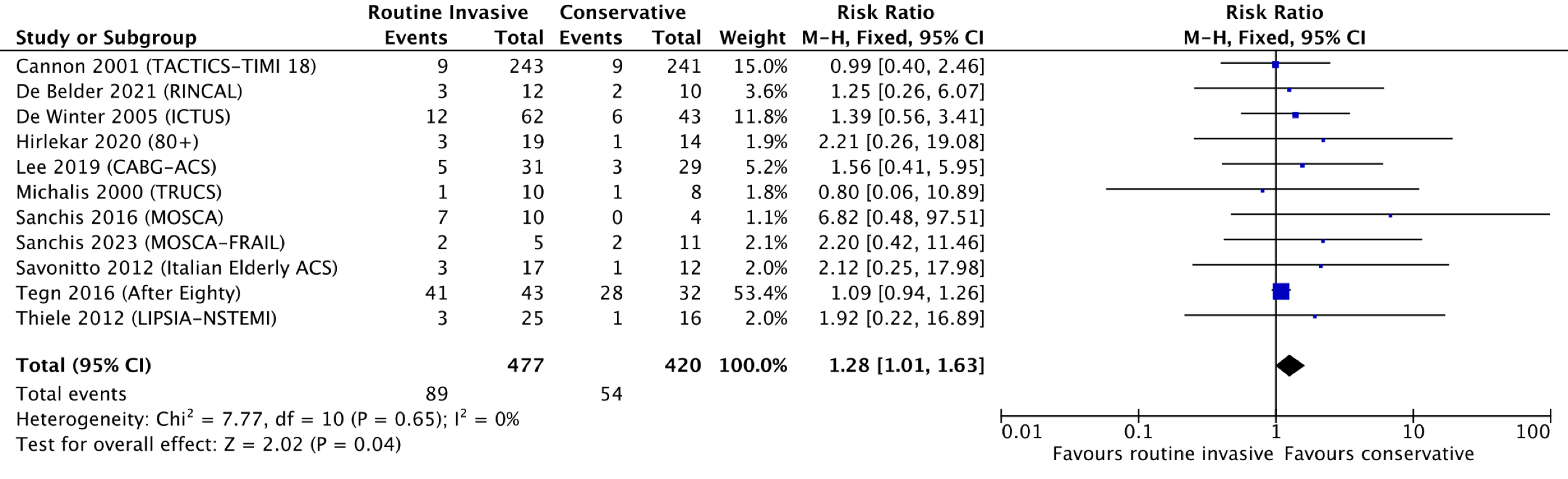
Figure S1: Effect on Mortality of Routine Invasive versus Conservative Strategy in CABG Patients Presenting with NSTE-ACS, fixed effects model

Table S3: Sensitivity Analysis of Trials by Elderly Patient Status

| Subgroup | Risk Ratio (95% CI) Random effects model | | | |
| --- | --- | --- | --- | --- |
|  | Mortality | Cardiac Mortality | Myocardial Infarction | Cardiac Hospitalisation |
| Non-Elderly (n=708)  TACTICS-TIMI 18; ICTUS; CABG-ACS; TRUCS; LIPSIA-NSTEMI | RR 1.25 (0.72-2.15) p=0.42 | RR 2.29 (0.39-13.34) p=0.36 | RR 0.88 (0.47-1.66, p=0.7) | RR 0.98 (0.69-1.40, p=0.91) |
| Elderly (n=189)  RINCAL; 80+; MOSCA; MOSCA-Frail; Italian Elderly ACS; After Eighty | RR 1.57 (0.76-3.25, p=0.23) | RR 1.14 (0.74-1.75, p=0.55) | R 1.00 (0.63-1.57, p=0.99) | RR 1.45 (0.85-2.47, p=0.17) |

Table S4: Sensitivity Analysis of Trials by Publication Date

| Subgroup | Risk Ratio (95% CI) Random effects model | | | |
| --- | --- | --- | --- | --- |
|  | Mortality | Cardiac Mortality | Myocardial Infarction | Cardiac Hospitalisation |
| Pre-2012 (n=677)  TACTICS-TIMI 18; ICTUS; TRUCS; Italian Elderly ACS; LIPSIA-NSTEMI | RR 1.24 (0.70-2.21) p=0.45 | RR 1.68 (0.35-8.09) p=0.52 | RR 1.00 (0.45-2.25) p=0.99 | RR 1.01 (0.70-1.44) p=0.97 |
| Post 2012 (n=220)  RINCAL; 80+; CABG-ACS; MOSCA; MOSCA-Frail; After Eighty | RR 1.47 (0.78-2.76) p=0.24 | RR 1.02 (0.67-1.55), p=0.94 | RR 0.88 (0.60-1.28) p=0.50 | RR 1.31 (0.78-2.19), P=0.31 |

Table S5: Cardiac Death Definitions

| **Study** | **Cardiac Death Definitions** |
| --- | --- |
| De Belder 2021 (RINCAL) | Cardiovascular Death: includes sudden cardiac death, death due to acute MI, death due to heart failure of cardiogenic shock, death due to stroke or other cardiovascular cause. And all death with undetermined cause. |
| Lee 2019 (CABG-ACS) | Cardiovascular death: Death from a cardiovascular cause and any death of unknown cause. |
| Sanchis 2016 (MOSCA) | Cardiac Death: Death from known cardiac cause and any death without another known cause |
| Sanchis 2023 (MOSCA-Frail) | Cardiac death: Death due to cardiac cause. Unwitnessed death and death of unknown cause were considered cardiac death. |
| Savonitto 2012 (Italian Elderly ACS) | Cardiovascular death defined as sudden death, death due to MI, stroke, pulmonary embolism, procedure-related death, as well as unwitnessed death. |
| Tegn 2015 (After Eighty) | Cardiovascular mortality: Death from a known cardiovascular cause and any death without another known cause |
| Thiele 2012 (LIPSIA-NSTEMI) | Cardiac death: Death from a known cardiac cause and any death without another known cause |

Figure S2: Effect on Cardiac Mortality of Routine Invasive versus Conservative Strategy in CABG Patients Presenting with NSTE-ACS, random effects model

Figure S3: Effect on Cardiac Mortality of Routine Invasive versus Conservative Strategy in CABG Patients Presenting with NSTE-ACS, fixed effects model

Table S6: Myocardial Infarction Definitions

| **Study** | **Myocardial Infarction Definitions** |
| --- | --- |
| Cannon 2001 (TACTICS-TIMI 18) | Non-fatal MI. TIMI definition (Antman 1999^36^)   1. CKMB above ULN (if PCI <24hr prior then ≥3x, if CABG <24hr prior then ≥5x) and increased >50% over previous. 2. New Q waves in ≥2 contiguous leads or new LBBB |
| De Belder 2021 (RINCAL) | New episode of cardiac chest pain associated with a rise in troponin exceeding ULN  Periprocedural MI=troponin >20% above baseline when it was above ULN and stable or falling |
| De Winter 2005 (ICTUS) | Universal definition (Thygesen 2007^37^)  Spontaneous: CKMB >ULN in setting of myocardial ischaemia  PCI related: CKMB x3 ULN  CABG related: CKMB x5ULN plus new Q wave/LBBB |
| Hirlekar 2020 (80+) | Spontaneous: Chest pain >10min with elevated cardiac troponin levels.  PCI related: MI within 24hours of PCI with troponin >3ULN |
| Lee 2019 (CABG-ACS) | Third Universal Definition of MI (Thygesen 2012^38^)  Troponin >ULN with one of symptoms of ischaemia, new ST changes/LBBB/Q waves, imaging evidence RWMA, thrombus on angiography  PCI related: Troponin >5x ULN if normal baseline or rise >20% if elevated and falling  CABG related: Troponin x10 ULN |
| Michalis 2000 (TRUCS) | Two out of three of:   1. Typical chest pain 2. Diagnostic ECG (mainly new Q wave) 3. CKMB >ULN (spontaneous), 1.5xULN (PCI related) |
| Sanchis 2016 (MOSCA) | Third Universal Definition of MI^38^ |
| Sanchis 2023 (MOSCA-Frail) | Third Universal Definition of MI^38^ |
| Savonitto 2012 (Italian Elderly ACS) | Universal Definition of MI^37^ |
| Tegn 2015 (After Eighty) | Universal definition of MI^37^ |
| Thiele 2012 (LIPSIA-NSTEMI) | Universal Definition of MI^37^  In hospital re-MI defined by new Q waves or ST elevation in 2 contiguous leads with >20mins symptoms or CKMB>5xULN/if CKMB >5xULN at baseline then>50% increase |

Figure S4: Effect on Myocardial Infarction of Routine Invasive versus Conservative Strategy in CABG Patients Presenting with NSTE-ACS, fixed effects model

Figure S5: Effect on Mortality or MI of Routine Invasive versus Conservative Strategy in CABG Patients Presenting with NSTE-ACS, random effects model

Table S7: Cardiac Hospitalisations Definitions

| **Study** | **Hospitalisations Reported as Outcome** |
| --- | --- |
| Cannon 2001 (TACTICS-TIMI 18) | Repeat Hospitalisation for ACS |
| De Belder 2021 (RINCAL) | Hospital Readmission for NSTEMI |
| Hirlekar 2020 (80+) | Hospitalisation for new AF or heart failure |
| Lee 2019 (CABG-ACS) | Hospitalisation for Heart Failure |
| Sanchis 2016 (MOSCA) | Readmission for Acute Heart Failure |
| Sanchis 2023 (MOSCA-Frail) | Readmission for ACS, Heart Failure, other cardiac reasons |
| Savonitto 2012 (Italian Elderly ACS) | Repeat hospital stays for cardiovascular causes |
| Thiele 2012 (LIPSIA-NSTEMI) | Unstable Angina |

Figure S6: Effect on Cardiac Hospitalisation of Routine Invasive versus Conservative Strategy in CABG Patients Presenting with NSTE-ACS, random effects model


Figure S7: Effect on Cardiac Hospitalisation of Routine Invasive versus Conservative Strategy in CABG Patients Presenting with NSTE-ACS, fixed effects model

Table S8: Non-CABG patient’s outcomes

|  | Mortality | | Myocardial Infarction | |
| --- | --- | --- | --- | --- |
| Included Patients | Random effects | Fixed effects | Random effects | Fixed effects |
| Non-CABG patients from trials in this meta-analysis (n=4,810) | RR 0.94 (0.81-1.09)  P=0.40, I^2^=17% | RR 0.94 (0.84-1.05)  P=0.29 | RR 0.88 (0.68-1.12)  P=0.30, I^2^=48% | RR 0.87 (0.74-1.02)  P=0.08 |
| Non-CABG patients from trials in this meta-analysis and TIMI IIIb, FRISC II, RITA 3 and VINO (n=10,681) | RR 0.87 (0.74-1.02)  P=0.09, I^2^=34% | RR 0.89 (0.80-0.99)  P=0.04 | RR 0.84 (0.71-1.00)  P=0.05, I^2^=38% | RR 0.84 (0.74-0.94)  P=0.004 |

Figure S8: Funnel Plot for Meta-Analysis of Mortality

Figure S9: Funnel Plot for Meta-Analysis of Cardiac Mortality

Figure S10: Funnel Plot for Meta-Analysis of MI

Figure S11: Funnel Plot for Meta-Analysis of Cardiac Hospitalisation

Table S9: Egger’s Test for the Explored Outcomes

| Outcome | Intercept | P Value |
| --- | --- | --- |
| All-cause Mortality | 0.19 | 0.384 |
| Cardiac Mortality | -0.29 | 0.386 |
| Myocardial Infarction | 0.067 | 0.057 |
| Cardiac Hospitalisation | 0.009 | 0.858 |

**Search Strategy-Cochrane Library**

Search Name: Kelham search

Date Run: 16/05/2022 11:03:56

Comment:

| ID | Search | Hits |
| --- | --- | --- |
| #1 | ((((acute NEXT coronary NEXT syndrome*) or (Non NEXT ST NEXT Elevat* NEXT Myocardial NEXT Infarction*) or "unstable angina") NEAR/3 (therap* or treat* or drug* or surg* or manage* or invasive or angioplasy or pci or conservative))):ti (Word variations have been searched) | 648 |
| #2 | ((((acute NEXT coronary NEXT syndrome*) or (Non NEXT ST NEXT Elevat* NEXT Myocardial NEXT Infarction*) or "unstable angina") NEAR/3 (therap* or treat* or drug* or surg* or manage* or invasive or angioplasy or pci or conservative))):ab (Word variations have been searched) | 931 |
| #3 | MeSH descriptor: [Acute Coronary Syndrome] explode all trees and with qualifier(s): [diet therapy - DH, drug therapy - DT, surgery - SU, therapy - TH] | 1286 |
| #4 | MeSH descriptor: [Non-ST Elevated Myocardial Infarction] this term only and with qualifier(s): [diet therapy - DH, drug therapy - DT, surgery - SU, therapy - TH] | 75 |
| #5 | MeSH descriptor: [Angina, Unstable] explode all trees and with qualifier(s): [diet therapy - DH, drug therapy - DT, surgery - SU, therapy - TH] | 760 |
| #6 | #1 or #2 or #3 or #4 or #5 with Publication Year from 1992 to 2022, with Cochrane Library publication date Between Jan 1992 and Jun 2022, in Trials | 2899 |

**Search Strategy-Embase and Medline**

Database: Embase <1974 to 2022 May 12>, Ovid MEDLINE(R) ALL <1946 to May 11, 2022>

Search Strategy:

--------------------------------------------------------------------------------

1 (("acute coronary syndrome*" or "Non ST Elevat* Myocardial Infarction*" or "unstable angina") adj4 (therap* or treat* or drug* or surg* or manage* or invasive or angioplasy or pci or conservative)).ti,ab. (18454)

2 Acute Coronary Syndrome/dh, dt, su, th or Non-ST Elevated Myocardial Infarction/dt, su, th or Angina, Unstable/dh, dt, rt, su, th (34941)

3 ("randomi?ed control* stud*" or "randomi?ed control* trial*").ti,ab. (570713)

4 1 or 2 (45967)

5 limit 4 to (english language and yr="1992 -Current" and randomized controlled trial) (4017)

6 3 and 4 (1535)

7 6 (1535)

8 limit 7 to (english language and yr="1992 -Current") (1469)

9 5 or 8 (5087)

10 acute coronary syndrome/dm, dt, rt, su, th or non ST segment elevation myocardial infarction/dm, dt, rt, su, th or unstable angina pectoris/dm, dt, rt, su, th (37107)

11 1 or 10 (47791)

12 limit 11 to (english language and randomized controlled trial and yr="1992 -Current") (4169)

13 3 and 11 (1577)

14 13 (1577)

15 limit 14 to (english language and yr="1992 -Current") (1509)

16 12 or 15 (5263)

17 9 use medall (2358)

18 16 use oemezd (2947)

19 17 or 18 (5305)

20 remove duplicates from 19 (4027)
